# Intolerance of uncertainty, psychopathology, and emerging adult sibling relationships

**DOI:** 10.1007/s12144-026-09413-x

**Published:** 2026-04-20

**Authors:** Derek D. Morgan, Monica J. Martin, Paul B. Ingram, Wonjung Oh, Christy R. Rogers

**Affiliations:** 1 Department of Psychological Sciences, University of Missouri, 200 Seventh St, Columbia, MO 65211 USA; 2 Department of Human Development and Family Sciences, Texas Tech University, Lubbock, USA; 3 Department of Psychological Sciences, Texas Tech University, Lubbock, USA

**Keywords:** Anxiety, Depression, Emerging Adult, Sibling, Substance Use, Uncertainty

## Abstract

Intolerance of uncertainty is often associated with emerging adult psychopathology, including depression, anxiety, and substance use behaviors. However, positive sibling relationships, which are known to support emerging adult mental health during stressful periods, may mitigate the stress related to uncertainty. Thus, this study investigated the role that sibling relationships may have between uncertainty and psychopathology. In a two-cohort longitudinal sample of emerging adults (*N* = 189, M*age* = 20.89), the moderating role of positive sibling relationship quality was examined between intolerance of uncertainty and later depression, anxiety, and substance use. Intolerance of uncertainty only predicted anxiety, while positive sibling relationship quality moderated the association between intolerance of uncertainty and later substance use, with low levels of sibling relationship quality showing a protective effect on substance use. Subsequent analyses revealed positive sibling relationship quality significantly moderated the association for marijuana only when the autoregressive effect was removed, resulting in greater marijuana use when sibling relationship quality was high. Positive sibling relationship quality also moderated the effect on health risk substance use, resulting in less substance use when sibling relationship quality was low. Findings underline the role that sibling relationships can play as both risk and resiliency factors for distinct emerging adult substance use behaviors. Sibling relationships continue to serve as significant sources of influence on emerging adult psychopathology.

## Introduction

Mental health typically worsens throughout emerging adulthood (Epstein et al., 2015; Finan et al., 2018). These declines can partially be explained by feelings of uncertainty during emerging adult transitions (Ben Salah et al., 2023). As emerging adults navigate new social demands and responsibilities in secondary education and career opportunities, they report greater stress toward future events (Arnett, 2015). Specifically, increased stress related to uncertain events can develop into feelings of intolerance of uncertainty (Carleton, 2016). While feelings of uncertainty can exacerbate mental health and substance usage during emerging adulthood (Oglesby et al., 2015), close family relationships, including those with siblings, may serve as protective factors against these challenges (Finan et al., 2018). Indeed, emerging adult sibling relationships can assist in reducing perceived stress and improving mental health (Milevsky, 2019). Although sibling relationships can support emerging adult mental health (Osman & Miranda, 2022), they are often overlooked as potential sources of support (Jensen et al., 2022). As limited research has examined potential resiliency factors against intolerance of uncertainty on psychopathology, current work suggests perceived social supports can protect against intolerance of uncertainty on depressive experiences (Ben Salah et al., 2023). Given the lack of clarity on types of relationships offer social supports that can mitigate the negative effects of intolerance of uncertainty on psychopathology, this study examined the potential role of sibling relationships to act as a source of social support in the link between intolerance of uncertainty and later psychopathology.

### Intolerance of uncertainty and psychopathology

Emerging adulthood, the developmental period from 18 to 29, spans the developmental period following adolescence and often serves as a transitionary period for individuals to explore their identities, interests, and more complex roles in adulthood (Arnett, 2015). Emerging adults experience greater instability in housing, education, and employment as they explore their adult roles and opportunities, and many report feeling *in-between*, such that they are not yet an adult nor are they still an adolescent (Arnett, 2015; Arnett & Schwab, 2012). Intolerance of uncertainty, a tendency to perceive uncertain situations as threatening and stressful, often develops due to growing concerns regarding experiences individuals face when provided little information (Carleton et al., 2007). Those who experience intolerance of uncertainty often report a reduced capacity to endure aversive situations (Carleton, 2016). Emerging adults are at risk for intolerance of uncertainty due to the tumultuous nature of emerging adulthood and evolving roles (student/new professional, child/adult, and individual/partner) which coalesce during this period (Arnett, 2015). Emerging adult intolerance of uncertainty also contributes to greater psychological distress (Ben Salah et al., 2023), and can worsen depression (Nekić & Mamić, 2019), anxiety (Panchyshyn et al., 2023), and substance use (Oglesby et al., 2015). An alarming number of emerging adults report mental health concerns, with one in three receiving a mental health diagnosis (Arnett & Schwab, 2012). Emerging adults face increased risks toward their psychopathology when they undergo greater instability (Arnett & Schwab, 2012), which can further delay attainment of adult roles (Arbona et al., 2021). To better support emerging adults, it’s crucial to understand how intolerance of uncertainty may contribute to their psychopathology.

#### Depression

Emerging adult depressive experiences peak in their early 20’s (Finan et al., 2018) and are characterized by greater sadness, despair, lower energy, and feelings of worthlessness. Life transitions can facilitate new stressors and depressive experiences for emerging adults (Arnett, 2015). Intolerance of uncertainty has been associated with greater depression (Nekić & Mamić, 2019), though recent work also suggests that this link may be due to the highly correlated nature of internalizing issues (Sahib et al., 2024). As depressive experiences can have lasting negative effects on emerging adult development, understanding risk factors that contribute to depression is key to support emerging adult mental health. To this end, examining whether intolerance of uncertainty might influence emerging adult depressive experiences over time is crucial to support their psychological development.

#### Anxiety

More than half of all emerging adults report anxiety disorders (Arnett & Schwab, 2012), which often encompass apprehension, feelings of fear and stress, and tension toward anticipated future misfortune. Transitions into college and full-time employment likely contributes to greater emerging adult psychological distress, and in turn can impede positive growth and later life stability. Intolerance of uncertainty can exaggerate emerging adult perceptions of stress and worry, as intolerance of uncertainty is linked with anxiety disorders (Arbona et al., 2021; Nekić & Mamić, 2019). Emerging adults who experience intolerance of uncertainty further report greater maladaptive coping (Panchyshyn et al., 2023), suggesting intolerance of uncertainty disrupts emerging adult ability to manage daily stressors. The prevalence of anxiety among emerging adults may be associated with intolerance of uncertainty, though limited research has examined these effects longitudinally.

#### Substance use

Substance use includes consumption, via inhaled, injected, or ingestion, of a variety of controlled legal or illicit substance for recreational use or misuse. Readily available substances, such as alcohol, marijuana, as well as other illicit substances, are known to have lasting negative effects on the development of emerging adults when misused (Stone et al., 2012). Intolerance of uncertainty can contribute to greater drinking behaviors in college students (Oglesby et al., 2015), as well as increased marijuana use (Bhati & Mathur, 2023). Substances with high addictive potential and significant negative health consequences—referred to as ‘health risk substances’ (i.e., certain opioids, amphetamines, and injectable drugs; Janik et al., 2017)—have also been linked to intolerance of uncertainty (Garami et al., 2017). However, the association between intolerance of uncertainty and substance use is less clear as greater intolerance of uncertainty can result in both high and low levels of substance use behaviors among emerging adults (Doruk et al., 2015; Oglesby et al., 2015). Examining the influence that intolerance of uncertainty can have on emerging adult substance use patterns overtime may then further delineate and clarify this association.

### Emerging adult sibling relationships

The family resilience framework suggests that close and supportive relationships with family, such as siblings, can reduce negative experiences and promote adaptive responses to stress (Walsh, 2016). Sibling relationships change and adapt across emerging adulthood and remain salient factors in emerging adult lives (Osman & Miranda, 2022). Moreover, siblings can influence emerging adult mental health through the perceived warmth and conflict they show one another. Indeed, warmth and support from sibling’s aid in lessening depressive symptoms for emerging adults (Milevsky, 2019). Additionally, sibling relationships in which emerging adults hold their siblings in higher esteem can reduce anxiety symptoms as emerging adults perceive greater sibling support and report less stress (Osman & Miranda, 2022). Furthermore, supportive sibling relationships can both encourage greater substance use and promote substance abstinence and safe use (Kothari et al., 2014). Nonetheless, sibling relationships often serve as stable resources during emerging adulthood, and may provide protective effects against stress, such as from intolerance of uncertainty, on psychopathology.

Furthermore, sibling socialization research has almost exclusively depicted top-down approaches, where younger siblings receive support from older siblings. However, bidirectional influences (older ↔ younger) are often suggested within the sibling relationship (Jensen et al., 2022). Bottom-up sibling influences from younger to older siblings in emerging adulthood may serve to be a source of resilience that older emerging adult siblings can utilize during stressful periods similar to how younger siblings utilize support from their older siblings (Milevsky, 2019). Indeed, prior work has shown that younger siblings can influence older sibling risk taking and substance use in adolescence (Whiteman et al., 2017), and may continue into emerging adulthood. Emerging adult older sibling psychopathology may benefit from positive relationships with their younger siblings, though these effects are yet to be seen. As such, examining bottom-up approaches may provide more clarity about the influence relationships with younger siblings can have on older sibling depression, anxiety, and substance use, especially in the context of intolerance of uncertainty.

Growing interest has begun to focus on the protective effects of siblings in emerging adulthood. However, the potential protective effects of sibling relationships have yet to be examined against intolerance of uncertainty and associated psychopathology for emerging adults. Emerging evidence suggests that social support may mitigate the impact that intolerance of uncertainty has on emerging adult psychopathology (Ben Salah et al., 2023). Though, social supports in prior work were broadly defined and not specific to any relationship that emerging adults may rely on during times of stress. As siblings often draw upon their relationship as sources of support during times of stress (Osman & Miranda, 2022), the sibling relationship may then help to lessen the negative effect that intolerance of uncertainty can have on their psychopathology. Moreover, limited research has investigated the effects of sibling relationships on older sibling mental health. Thus, we examined whether sibling relationships moderated the association between intolerance of uncertainty and later psychopathology for older siblings in emerging adulthood.

### Current study

Intolerance of uncertainty can serve as a risk factor for emerging adult psychopathology (Garami et al., 2017; Nekić & Mamić, 2019). Identifying protective factors to support psychopathology is important to alleviate emerging adult adverse experiences. Sibling relationships can offer adaptive support for emerging adult mental health concerns (Finan et al., 2018) and may serve as protective factors against stressful experiences, like intolerance of uncertainty. Younger adolescent siblings may provide benefits to older emerging adult siblings when sibling relationship quality is high. To enhance our understanding of emerging adult mental health and the effect of sibling relationships, this study seeks to examine (1) the effect of intolerance of uncertainty on later depression, anxiety, and substance use, and (2) the separate moderating effect of sibling relationship quality between intolerance of uncertainty and later depression, anxiety, and substance use. It is hypothesized that higher intolerance of uncertainty will be associated with greater depression, anxiety, and substance use later on. Sibling relationship quality is expected to moderate the association between intolerance of uncertainty and later psychopathology, such that higher quality sibling relationships will lessen the negative effects of intolerance of uncertainty on later emerging adult depression, anxiety, and substance use.

## Methods

### Participants

The present study used data from a two-cohort longitudinal study that included 939 emerging adult college students. A total of 189 emerging adults (M*age* = 20.89 years old; 170 women) completed two online surveys about their experiences and their younger adolescent sibling (M_age_ = 16.51 years old; 110 girls). College attending emerging adults in West Texas were included if they were between the ages of 18–25 and had a younger adolescent sibling who was at least 1 year younger (between the ages of 12–19). In the case that emerging adults had multiple younger siblings, they reported on the younger sibling closest in age. Emerging adult youngest siblings were excluded, as the study was focused on examining older sibling relationships to younger siblings. Emerging adults were recruited through university listservs and flyers shared in university courses during the fall semester of 2021 and 2022.

Emerging adults identified primarily as White (57.9%), as well as Hispanic/Latino (22.8%). A third of participants had only one sibling (33.3%), followed by two siblings (28.0%) and three siblings (16.9%). The remaining participants (21.8%) had four or more siblings. Most emerging adults (155, 82%) reported living less than 25% of the time with their parents and were solely responsible for their income. Average emerging adult income was less than $14,999, while the average parental income was between $75,000 and $89,999. Demographics are reported in Table 1.

**Table 1:** Emerging adult ethnicity, number of siblings, and participant/family total incom*e*

Variables	*N* * = 189 (%)*	
*Ethnicity*		
African or Black	13 (6.9%)	
American Indian or Alaskan Native	2 (1.1%)	
White	109 (57.9%)	
East Asian	3 (1.5%)	
Hispanic/Latino	43 (22.8%)	
South Asian	1 (0.5%)	
Other	17 (9.0%)	
*Number of Siblings*		
1	63 (33.3%)	
2	53 (28.0%)	
3	32 (16.9%)	
4	14 (7.4%)	
5	8 (4.2%)	
6	9 (4.7%)	
7	3 (1.6%)	
8	1 (0.5%)	
9 or more	6 (3.2%)	
*Income*	Participant (*n* = 155)	Family (*n* = 34)
$0 - $14,999	117 (75.4%)	1 (2.9%)
$15,000 - $29,999	20 (12.9%)	4 (11.7%)
$30,000 - $44,999	9 (5.8%)	2 (5.8%)
$45,000 - $59,999	—	1 (2.9%)
$60,000 - $74,999	2 (1.3%)	4 (11.7%)
$75,000 - $89,999	1 (0.6%)	4 (11.7%)
$90,000 - $99,999	—	1 (2.9%)
$100,000 - $119,999	2 (1.3%)	3 (8.8%)
$120,000 - $150,000	—	6 (17.6%)
$150,000 +	10 (2.5%)	8 (23.5%)

Family’s income reported if participants spent more than 50% of the year living with parents

### Procedures

A cross-sequential design was implemented to assess two cohorts of emerging adults during two waves of data collection across the academic year (fall, spring). A university online student research pool system was used to host participant enrollment. Emerging adults received course extra credit or a $10 Amazon gift card for their participation. Emerging adults completed Time 1 (T1) fall surveys from September to December and Time 2 (T2) spring surveys from February to May. These procedures were in accordance with the online research system to ensure that participating emerging adults were awarded extra credit on the last day of classes. All procedures were approved by the Institutional Review Board, and participants provided informed consent.

Cohort 1 participated during the 2021–2022 academic year (*N* = 455; 287 women; 25.1% retention) and cohort 2 participated during the 2022–2023 academic year (*N* = 485; 391 women; 15.4% retention), with a total of 189 (170 women) emerging adults retained for the second survey (T2) across both cohorts. Due to low retention rates commonly observed in longitudinal studies following COVID-19 (Yu et al., 2022), a second cohort was recruited to increase sample size and reduce potential cohort bias. This was the first study to launch a longitudinal component in the specific university research pool system used for this study. The new longitudinal study aspect, likely contributed to the low retention rates.

### Measures

#### T1 and T2 intolerance of uncertainty

Emerging adults reported on the Intolerance of Uncertainty Scale (Carleton et al., 2007). The 12-item measure examines the ability to tolerate uncertain events and their impact on problem solving skills. Items included statements such as, “Unforeseen events upset me greatly,” and, “When I am uncertain, I can’t function very well.” Items were scored on a 5-point Likert scale, ranging from 1 (*not at all*) to 5 e*ntirely*). Items were summed with higher scores indicating greater intolerance of uncertainty. The scale showed excellent internal reliability at T1 (α = 0.94) and T2 (α = 0.95).

#### T1 positive sibling relationship quality

Emerging adults reported on the Behavioral Affective Rating Scales (Rogers et al., 2018), a 12-item measure assessing perceptions of directional warmth and hostility. Emerging adults reported on their younger sibling’s affect and behavior toward them in the past month. Warmth was measured using items such as, “Lets me know they appreciate me, my ideas, or the things I do,” whereas hostility included items such as, “Ignore me when I try to talk to them.” Items ranged from 1 (*never*) to 7 (*always*), and were averaged into subscales of warmth (α = 0.88) and hostility (α = 0.91). Hostility items were reverse coded such that higher scores indicated lower hostility. Together, the warmth and reverse coded hostility items represent positive sibling relationship quality, which had good internal consistency (α = 0.82).

#### T1 and T2 depression

The Patient Health Questionnaire 9-Item (Kroenke et al., 2001) was used to measure depressive experiences in emerging adults at T1 and T2. Items on the PHQ-9 include those such as, “Feeling down, depressed, or hopeless,” and “Trouble concentrating on things, such as reading the newspaper or watching television”. Items are scored from 0 (*not at all*) to 3 (*daily*), with higher scores indicating more depressive experiences. Score ranges of five to nine indicate mild depression, while scores of 10 to 14 indicate moderate depression and clinical impairments (e.g., Morris et al., 2023). The PHQ-9 demonstrated excellent reliability (T1 α = 0.91; T2 α = 0.92).

#### T1 and T2 anxiety

Participants reported at T1 and T2 on their anxiety experiences over the last two weeks using the Generalized Anxiety Disorder 7-Item (Spitzer et al., 2006). Items included, “Feeling nervous, anxious, or on edge” and “Feeling afraid as if something awful might happen”. Response categories range from 0 (*not at all*) to 3 (*daily*) with higher scores indicating worse anxiety related experiences. In general, scores of 10 or above indicate moderate or worse levels of anxiety that may require clinical care (e.g., Spitzer et al., 2006). The GAD-7 had excellent internal consistency (T1 α = 0.95; T2 α = 0.95).

#### T1 and T2 substance use

Emerging adult substance use was captured at T1 and T2 using the Diagnostic and Statistical Manual of Mental Disorders (DSM) cross cutting symptom assessment (WHO ASSIST Working Group, 2002). The cross-cutting assessment is used to examine substance misuse over the last two weeks. A single item was used to assess marijuana use, while health risks substances (e.g., opioids, amphetamines, hallucinogens) were assessed using a combination of multiple items, such as, “Heroin,” and “Opioids.” Items ranged from 1 (*not at all*) to 5 (*nearly every day*) and showed great internal consistency (T1 α = 0.94; T2 α = 0.89).

Emerging adults additionally reported on their alcohol use frequency (Martin et al., 2019) to examine the degree to which emerging adults used alcohol over the past month. The three-item scale included types of alcohol use including, “Drink beer”, “Drink wine, wine cooler, other wine drinks”, and, “Drink hard liquor, such as bourbon, vodka, whiskey, or gin.” Items ranged from 1 (*never*) to 6 (e*veryday*) and showed satisfactory internal consistency (T1 α = 0.80; T2 α = 0.86).

#### Covariates

Age and gender of emerging adults and their younger sibling, as well as reported income and number of siblings, were included as covariates. Latent variables of previous reports of psychopathology (T1) were also included. Age in years was computed as a continuous variable by subtracting date of birth from the session date. Gender was coded as 1 (*women*) and 2 (*men*), and income was coded in $15,000 segments, from 1 ($0-$14,999), to 10 ($150,000+).

### Analytic plan

Attrition analyses were conducted on the full sample (*N* = 939) and subsample (*N* = 189) to determine if sample attrition affected results. There were no significant differences in T1 covariates or variables of interests, except for positive sibling relationship quality (t = −3.18, *p* <.001). This difference indicates that the subsample had a higher mean in positive sibling relationship quality *(M =* 5.43) compared to the full sample *(M* = 5.26).

All data analyses were conducted using *Mplus* 8.7 (Muthén & Muthén, 2022). Maximum likelihood (ML) estimation was used to provide less biased estimates and greater inclusion of missing data. Random parceling of scale items was used to stabilize parameter estimates and reduce random error for observed latent constructs (Little et al., 2002). Intolerance of uncertainty, depression, anxiety, substance use, and positive sibling relationship quality were created into latent continuous variables using parceling. The latent variable for substance use included the cross-cutting assessment and the alcohol use frequency scale. Confirmatory factor analysis (CFA) was conducted to ensure item measurement fit the data well. Further, it provided bivariate correlations among study constructs to verify the value of testing the proposed model.

To test hypothesis 1, a single structural equation model (SEM) was estimated with T1 intolerance of uncertainty predicting later depression, anxiety, and substance use given the comorbidity and covariance shared between depression, anxiety, and substance use. Latent variables of T1 depression, anxiety, and substance use, as well as T2 intolerance of uncertainty, were also included in this model with autoregressive paths estimated. Next, covariates of emerging adult and younger sibling age and gender, participant income, and number of siblings were added to the model. Conventional cutoffs were used for several model fit indices: a comparative fit index (CFI) and Tucker-Lewis index (TLI) of 0.95 or more, and a root mean square error of approximation (RMSEA) below 0.08 (Brown, 2015). To test hypothesis 2, separate moderation SEMs were computed to estimate T1 positive sibling relationship quality as a moderator between pathways for intolerance of uncertainty on later depression, anxiety, and substance use. Simple slope analyses were computed in *Mplus* for significant interaction coefficients at one standard deviation above and below the mean.

## Results

### Descriptive statistics of latent variables

All factor loading values were in the expected direction, of acceptable magnitude, and statistically significant, affirming the usefulness of the variables used to measure the latent constructs. The CFA fit the data well (χ2 = 986.27, df = 465, RMSEA = 0.07, 95% CI [0.07, 0.08], SRMR = 0.07, CFI = 0.90, TLI = 0.89). Pearson correlations (See Table 2) showed that T1 intolerance of uncertainty was significantly and positively correlated with T2 depression (*r* =.33) and anxiety (*r* =.48), but not substance use (*p* =.36), suggesting intolerance of uncertainty was strongly associated with depression and anxiety, evidencing large effects. T1 positive sibling relationship quality was negatively and significantly correlated with T1 intolerance of uncertainty (*r* = −.16), as well as T2 depression (*r* = −.21) and substance use (*r* = −.28), suggesting that greater positive sibling interactions associate with less psychopathology in general. Notably, internalizing issues of depression and anxiety were strongly correlated (*r* =.84 −.85).

**Table 2:** Pearson correlations of variables of interest

Measures	1	2	3	4	5	6	7	8	9	10	11	12	13	14	15
1. T1 Intolerance of Uncertainty	—														
2. T1 Positive Sibling Relationship Quality	− 0.16*	—													
3. T1 Depression	0.47***	− 0.11	—												
4. T1 Anxiety	0.64***	− 0.08	0.85***	—											
5. T1 Substance Use	0.06	− 0.26**	0.28***	0.18*	—										
6. T1 Marijuana Use	0.16*	− 0.19**	0.17*	0.18*	—	—									
7. T1 Health Risk Substances	0.09	− 0.25**	0.23**	0.11	—	0.27***	—								
8. T1 Alcohol Use	0.00	− 0.11	0.22**	0.18*	—	0.27***	0.41***	—							
9. T2 Intolerance of Uncertainty	0.59***	− 0.15*	0.27***	0.39***	0.08	0.24***	0.06	0.09	—						
10. T2 Depression	0.33***	− 0.21**	0.62***	0.55***	0.17*	0.12	0.11	0.16*	0.44***	—					
11. T2 Anxiety	0.48***	− 0.12	0.51***	0.59***	0.13	0.16*	0.07	0.14*	0.64***	0.84***	—				
12. T2 Substance Use	− 0.07	− 0.28***	0.18*	0.10	0.61***	—	—	—	0.14	0.37***	0.20**	—			
13. T2 Marijuana Use	0.12	− 0.18*	0.34***	0.26***	—	0.71***	0.17*	0.21**	0.24***	0.35***	0.30***	—	—		
14. T2 Health Risk Substances	− 0.06	− 0.26***	0.12	0.06	—	0.23**	0.46***	0.33***	0.13	0.28***	0.14	—	0.31***	—	
15. T2 Alcohol Use	− 0.04	− 0.22**	0.17*	0.10	—	0.27***	0.35***	0.60***	0.07	0.32***	0.19***	—	0.33***	0.56***	—

Abbreviations: *T1* Time 1, *T2* Time 2. All variables are latent except for T1 and T2 marijuana use, health risk substances, and alcohol use. Correlations between substance use and marijuana, health risk, and alcohol left blank as the substance use latent variable contains these measures

*p* <.05*, *p* <.01**, *p* <.001***

### Uncertainty, sibling relationships, and psychopathology

The structural model fit the data well (RMSEA = 0.07, 95% CI [0.06, 0.08], SRMR = 0.06, CFI = 0.91, TLI = 0.90), findings shown in Table 3, Model 1. Intolerance of uncertainty significantly predicted T2 anxiety (β = 0.19, *p* =.01), such that greater emerging adult experiences of intolerance of uncertainty at T1 were associated with greater experiences of T2 anxiety. However, T1 intolerance of uncertainty did not predict T2 depression (β = 0.07, *p* =.33) or substance use (β = − 0.10, *p* =.11). T1 positive sibling relationship quality did predict less T2 depression (β = − 0.13, *p* =.04), but was not associated with T2 anxiety (β = − 0.03, *p* =.52), or substance use (β = − 0.10, *p* =.06). Autoregressive paths of variables were all significant. Among covariates, emerging adult gender was negatively and significantly associated with T2 substance use (β = − 0.15, *p* =.01), such that identifying as a woman associated with lower overall substance use at follow up. Younger sibling age was also negatively associated with T2 depression (β = 0.14, *p* =.03), anxiety (β = 0.15, *p* =.01), and intolerance of uncertainty (β = 0.13, *p* =.03), such that having an early, compared to an middle or older, adolescent younger sibling associated with less depression, anxiety, and intolerance of uncertainty later on.

**Table 3:** Main and interactive effects on emerging adult psychopathology

Model 1	T2 Depression	T2 Anxiety	T2 Substance use	T2 Intolerance of uncertainty		
T1 Intolerance of Uncertainty	0.07	0.19**	− 0.10	0.59***		
T1 Positive Sibling Relationship Quality	− 0.13*	− 0.03	− 0.12	—		
T1 Income	− 0.05	− 0.05	− 0.04	− 0.04		
T1 Number of Siblings	− 0.01	− 0.05	− 0.02	− 0.01		
T1 Age	− 0.01	0.05	− 0.04	0.00		
T1 YS Age	− 0.14*	− 0.13*	0.01	− 0.14*		
T1 Women vs. Men	0.08	0.05	− 0.15*	− 0.09		
T1 YS Girls vs. Boys	0.04	0.02	0.01	0.00		
T1 Control	0.52***	0.44***	0.56***	—		
Intolerance of Uncertainty X Positive Sibling Relationship Quality	− 0.01	− 0.04	0.10**	—		
Model 2	T2 Depression	T2 Anxiety	T2 Marijuana Use	T2 Health Risks Substances	T2 Alcohol Use	T2 Intolerance of Uncertainty
T1 Intolerance of Uncertainty	0.07	0.19**	0.09	− 0.11	− 0.03	0.62***
T1 Positive Sibling Relationship Quality	− 0.13*	− 0.03	− 0.16*	− 0.14*	− 0.15*	—
T1 Income	− 0.05	− 0.05	− 0.12	− 0.04	0.01	− 0.04
T1 Number of Siblings	− 0.01	− 0.05	0.11	0.04	− 0.12	− 0.01
T1 Age	− 0.01	0.05	0.01	− 0.03	− 0.01	0.00
T1 YS Age	− 0.13*	− 0.13*	0.05	0.04	− 0.03	− 0.13*
T1 Women vs. Men	0.09	0.05	− 0.07	− 0.12	− 0.18**	− 0.09
T1 YS Girls vs. Boys	0.04	0.02	− 0.03	0.04	− 0.02	0.00
T1 Control	0.50***	0.42***	—	0.43***	0.53***	—
Intolerance of Uncertainty X Positive Sibling Relationship Quality	0.01	− 0.04	− 0.22*	0.12***	− 0.09	—

Significant effects are bolded. All values are standardized estimates. Gender coded as 1 = Woman/girl, 2 = Man/boy. Abbreviations: T1 = Time 1; T2 = Time 2; YS = Younger Sibling. Autoregressive effect of T1 marijuana on T2 not included in final model due to high collinearity

*p* <.05*, *p* <.01**, *p* <.001***

**Fig. 1:**
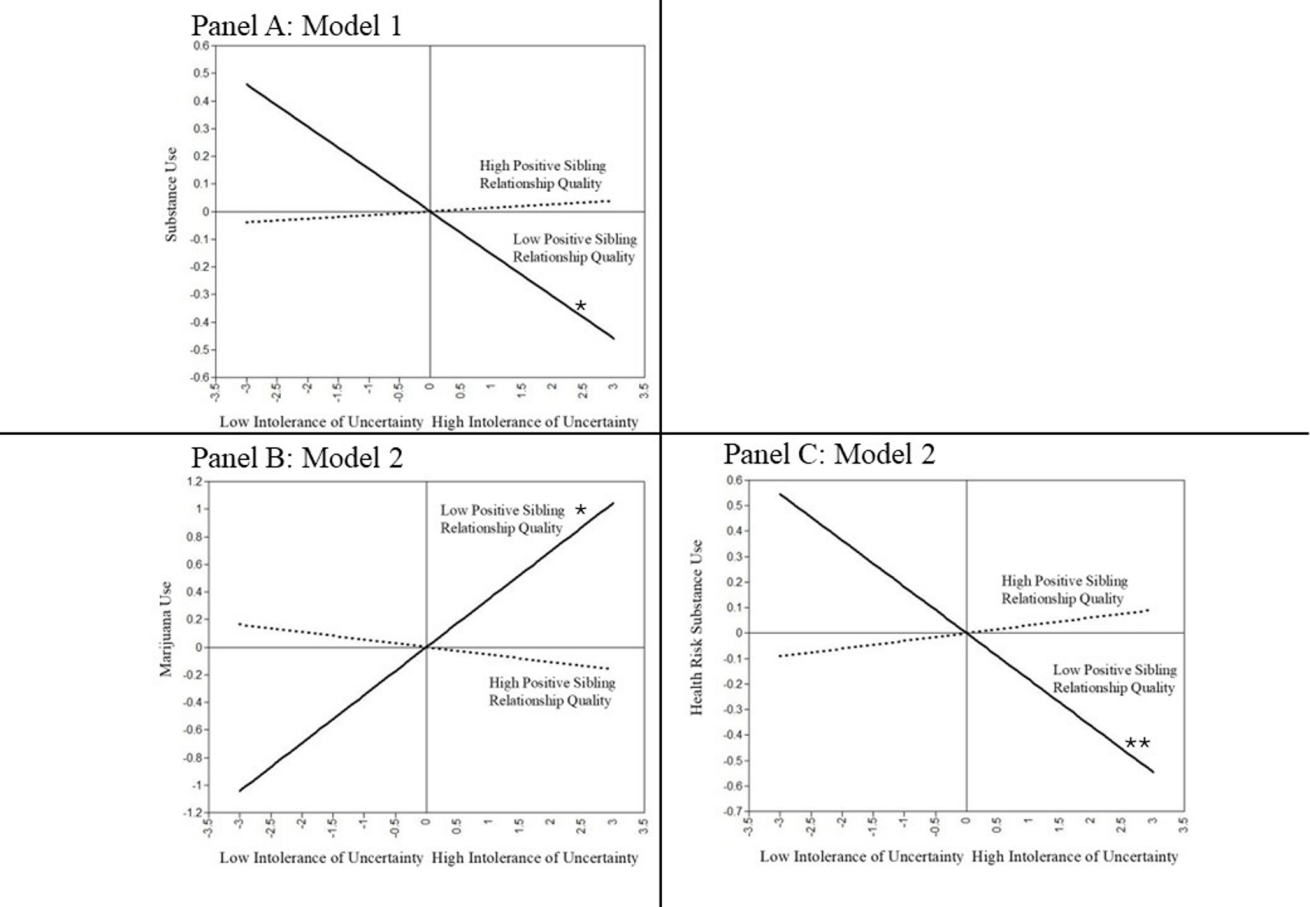
Interaction between intolerance of uncertainty and positive sibling relationship quality predicting later substance use (Panel **A**), marijuana use (Panel **B**) and later health risk substance use (Panel **C**)

Next, positive sibling relationship quality was tested in separate models as a moderator between T1 intolerance of uncertainty and later depression, anxiety, and substance use. Positive sibling relationship quality did not significantly moderate the association between T1 intolerance of uncertainty and T2 depression (β = − 0.01, *p* =.90) nor T2 anxiety (β = − 0.04, *p* =.27). There was a significant moderating effect of positive sibling relationship quality between T1 intolerance of uncertainty and T2 substance use (β = 0.10, *p* =.005; see Fig. 1, Panel A). Simple slope analyses showed a significant negative association between intolerance of uncertainty and later substance use for participants who reported low positive sibling relationship quality (*t* = −3.13, *p* =.002), suggesting that emerging adults with less positive sibling relationships engage in lower substance use following greater instances of intolerance of uncertainty. However, the association between intolerance of uncertainty and positive sibling relationship quality was not significant for those reporting high positive sibling behaviors (*t* = 0.99, *p* =.31), indicating that substance use was not associated with previous intolerance of uncertainty for emerging adults with more positive sibling relationships.

### Delineating substance use

Ad hoc analyses were conducted to more closely examine the interaction between intolerance of uncertainty and positive sibling relationship quality and their influence on specific emerging adult substance use behaviors. A CFA model was estimated with marijuana, health risk substances, and alcohol use measured as (1) manifest variables and (2) as latent variables. The model utilizing manifest variables fit the data better (χ2 = 793.91, df = 429, RMSEA = 0.06, 95% CI [0.06, 0.07], SRMR = 0.06, CFI = 0.92, TLI = 0.91) than the model including substance use variables as latent factors (χ2 = 1093.56, df = 453, RMSEA = 0.08, 95% CI [0.08, 0.09], SRMR = 0.10, CFI = 0.89, TLI = 0.88), thus the manifest model was selected in subsequent analyses. Notably, there was a high and significant correlation between T1 and T2 marijuana use (*r* =.70, *p* <.001). To better control for this effect, the autoregressive path of T1 to T2 marijuana use was not included in the below models, with any significant changes to results indicated.

The structural model of T1 intolerance of uncertainty predicting T2 depression, anxiety, marijuana, health risk substances, and alcohol use with positive sibling relationship quality and autoregressive paths also fit the data well (RMSEA = 0.07, 95% CI [0.07, 0.08], SRMR = 0.07, CFI = 0.90, TLI = 0.88), correlations are shown in Table 2 and findings are presented in Table 3, Model 2. Consistent with the prior structural model, T1 intolerance of uncertainty significantly predicted T2 anxiety (β = 0.19, *p* =.009), but not T2 depression (β = 0.07, *p* =.27), marijuana use (β = 0.09, *p* =.20), health risk substances (β = − 0.11, *p* =.08), or alcohol use (β = − 0.03, *p* =.56). This suggests that greater intolerance of uncertainty predicted later anxiety issues, even when controlling for other internalizing and externalizing behaviors. However, positive sibling relationship quality (T1) did predict lower T2 depression (β = − 0.13, *p* =.04), marijuana use (β = − 0.16, *p* =.02), and health risk substance use (β = − 0.14, *p* =.03), and alcohol use (β = − 0.15, *p* =.02), but was not associated with later anxiety (β = − 0.03, *p* =.53). All autoregressive paths were significant. Among covariates, participant gender was significantly associated with alcohol use (β = − 0.18, *p =*.002), suggesting that those who identified as women reported lower alcohol and health risk substance use. Additionally, younger sibling age was negatively associated with T2 depression (β = − 0.13, *p* =.04), anxiety (β = − 0.13, *p* =.04), and intolerance of uncertainty (β = − 0.13, *p* =.03), suggesting those with early adolescent siblings reported less impactful internalizing issues later on. When the autoregressive path of T1 to T2 marijuana use was included, positive sibling relationship quality no longer predicts later marijuana use (*p =*.49), though no other effects were influenced.

Next, separate moderation models were tested to examine the interaction effect of T1 intolerance of uncertainty and positive sibling relationship quality on T2 marijuana use, health risks substances, and alcohol use. Initial models accounting for the autoregressive path of T1 marijuana use, showed a nonsignificant moderation effect (β = − 0.02, *p* =.77). However, sensitivity analyses were run with T1 marijuana use excluded from the model (see Table 3, Model 2). Positive sibling relationship quality moderated the association between T1 intolerance of uncertainty and T2 marijuana use (β = − 0.22, *p* =.03; see Fig. 1 Panel B), when baseline marijuana use was excluded. Simple slope analyses showed a significant positive association between intolerance of uncertainty and later marijuana use for those with low positive sibling relationship quality (*t* = 2.42, *p* =.01), suggesting that less positive relationships with younger siblings exacerbated the effect of intolerance of uncertainty on later use marijuana use. On the other hand, the interaction between intolerance of uncertainty and high positive sibling relationship quality was not significant (*t* = − 0.79, *p* =.42), suggesting that emerging adults with more positive interactions with younger siblings experienced an ameliorating effect between experiences of intolerance of uncertainty and later marijuana use.

Positive sibling relationship quality also significantly moderated the association between T1 intolerance of uncertainty and T2 health risk substances (β = 0.12, *p* <.001; see Fig. 1 Panel C). Simple slope analyses showed a significant negative association between intolerance of uncertainty and later health risk substance use for those who reported low positive sibling relationship quality (*t* = −4.10, *p* <.001), suggesting that emerging adults with poorer sibling relationships show lower use of later health risks substances following high intolerance of uncertainty. However, the association between intolerance of uncertainty and health risk substances was not significant for emerging adults who experienced more positive sibling relationships (*t* = 1.47, *p* =.14). These moderation results parallel those found for the composite substance use outcome variable.

Finally, positive sibling relationship quality was examined as a moderator between T1 intolerance of uncertainty and T2 alcohol use. However, positive sibling relationship quality did not significantly moderate this association (β = − 0.09, *p* =.10). The moderating effect of positive sibling relationship quality was also re-examined for T2 depression (β = 0.01, *p* =.79) and anxiety (β = − 0.04, *p* =.29) in this model, but was not significant.

## Discussion

This study aimed to examine the effect of intolerance of uncertainty on later emerging adult depression, anxiety, and substance use, while examining the potential protective effect of emerging adult positive sibling relationship quality. Despite the range of difficulties that emerging adults face with intolerance of uncertainty as they navigate new adult roles and environments, limited research has examined potential protective factors to support folks during this transition (Ben Salah et al., 2023). Further, few prior studies account for the shared variance between depression, anxiety, and substance use when examining the effect of intolerance of uncertainty. In this study, two cohorts of emerging adults were followed, each over two college semesters, reporting on their mental health experiences, intolerance of uncertainty, and their relationships with their younger adolescent siblings. Findings showed intolerance of uncertainty predicted later emerging adult anxious symptoms but not depressive symptoms or substance use. Sibling relationship quality modulated the association between intolerance of uncertainty on later emerging adult substance use behaviors, particularly for health risk substances, as well as marijuana use when not accounting for baseline use. These findings provide support for intolerance of uncertainty as a risk factor for emerging adult psychopathology, as well as the unique effect sibling relationships have on emerging adult substance use behaviors.

### Uncertainty and depression

Contrary to our expectations, intolerance of uncertainty did not predict later depression for emerging adults. Much of the existing work suggests an association between intolerance of uncertainty and depressive symptoms is cross-sectional (Nekić & Mamić, 2019), likely as intolerance of uncertainty can heighten concurrent depressive symptoms but may fail to influence these symptoms over time (Sahib et al., 2024). Additionally, though the sample was majority women, who typically report greater depressive experiences than men (Essau et al., 2010), the association between intolerance of uncertainty and depression was not found despite research suggesting that intolerance of uncertainty has a stronger effect on women’s depressive experiences (Panchyshyn et al., 2023). Intolerance of uncertainty likely interacts with processes that are integrated in both depression and anxiety symptomology. Internalized issues often overlap in symptomology, but by accounting for prior depressive experiences research can better delineate these constructs and identify accurate predictors. Research should consider how intolerance of uncertainty influences similar processes in depression and anxiety, to better understand which symptoms are most impacted to better separate apart these influences (Nekić & Mamić, 2019). In addition, investigating how intolerance of uncertainty changes over time alongside depressive experiences can better inform our understanding of these processes.

Perceived social support has been shown to buffer the negative effects of intolerance of uncertainty from developing into depressive symptoms (Ben Salah et al., 2023), and positive sibling relations were associated with reduced later depression. However, emerging adult perceptions of positive relationships with their younger adolescent siblings were not found to moderate this pathway. The current findings showed that there was not a link between intolerance of uncertainty and later depressive experiences, and the effect of prior sibling warmth on depression was relatively small. Though sibling warmth may be helpful against depression in some cases (Milevsky, 2019), the interaction between sibling warmth and intolerance of uncertainty likely would not offer protections given intolerance of uncertainty was not associated with depressive experiences (Sahib et al., 2024). However, as the pressures and demands of new adult roles surpass the potential benefits of younger sibling warmth on depression, examining how siblings support one another during difficult times may be beneficial. Thus, research should continue to examine the effect that younger siblings can have on older sibling mental health.

### Uncertainty and anxiety

Intolerance of uncertainty is well grounded within anxiety frameworks (Carleton, 2016; Nekić & Mamić, 2019). Emerging adult experiences with intolerance of uncertainty may be important markers for identification of anxiety symptoms (Panchyshyn et al., 2023), especially as it pertains to college students. Emerging adults experience multiple transitions and changing roles regarding their education, careers, and social lives that can increase stress and worry regarding their potential futures that can lead to greater anxiety (Arbona et al., 2021). The resulting stress may accumulate into an intolerance of uncertainty which continues to negatively impact their daily mental health through anxiety, likely lessening their ability to manage their daily lives and hindering positive future development and prolonging emerging adulthood. As intolerance of uncertainty is a clear risk factor for anxiety, future research should consider how intolerance of uncertainty impacts emerging adults daily functioning through greater anxious experiences to better determine the long-term impacts on emerging adults.

Social support has been suggested to alleviate the negative effect of intolerance of uncertainty on anxiety (Ben Salah et al., 2023), though this study found that positive sibling relationship quality did not serve as a protective factor. As sibling relationships can provide adaptive benefits for emerging adults experiencing anxious symptoms (Osman & Miranda, 2022), the link between intolerance of uncertainty and anxiety may be stronger than the support perceived from younger siblings. Communication between siblings may potentially play a strong role given the differences in-person and virtual supports between adolescent and emerging adult siblings can have on mental health (Lindell et al., 2015). Future research should consider multiple relationships (i.e., friends, parents), as well as the effect of in-person and virtual supports, as potential buffers against intolerance of uncertainty and anxiety for emerging adults.

### Uncertainty and substance use

Intolerance of uncertainty did not directly predict substance use in emerging adults, possibly due to its strong association with depression and anxiety (Carleton, 2016). Given that emerging adults may seek out substance use to alleviate their depression or anxiety symptoms (Schulte & Li, 2022), controlling for both depression and anxiety, as well as accounting for prior substance use may have lessened the effect intolerance of uncertainty has on later substance use. In addition, as women report lower substance use compared to men (Stone et al., 2012), it’s likely that the effect of intolerance of uncertainty on substance use may have been more difficult to detect in the current sample. Future work would benefit from examining gender differences in the association between intolerance of uncertainty and substance use among more balanced samples, as well as for those that report consistent use.

The quality of positive relationships with younger siblings moderated the association between intolerance of uncertainty and later substance use. As intolerance of uncertainty is associated with varying levels of substance use (Doruk et al., 2015; Oglesby et al., 2015), social relationships, including those with siblings, may be a key mechanism through which intolerance of uncertainty influences substance use behaviors. Experiences of lower relationship quality with younger siblings showed a negative association between intolerance of uncertainty and later substance use, suggesting that less positive relations with younger siblings may result in reduced emerging adult substance usage following greater uncertainty. A lack of support from younger siblings may lead to negative perceptions for recreational substance use when intolerance of uncertainty is high and thus reduce emerging adult substance usage (Tucker et al., 2020). Indeed, when siblings’ relationship quality is low, adolescent and emerging adult siblings often report lower substance usage (Whiteman et al., 2017). This may be due to siblings spending less time with one another and thus having less opportunities to pass along or encourage negative substance use behaviors. These findings suggest that sibling relationships continue to influence behaviors well into emerging adulthood, with lacking sibling support potentially reducing ones’ reliance on substance use as a coping mechanism during periods of greater stress.

#### Marijuana use

Prior research suggests that greater experiences of intolerance of uncertainty associate with greater marijuana use (Bhati & Mathur, 2023); however, a direct effect was not found in this study. Marijuana use can be influenced by social factors (Epstein et al., 2015), such that positive interactions with close others can lead to initiation of marijuana use through deviancy training. As the current study examined marijuana, health risk substances, and alcohol simultaneously, as well as depression and anxiety concurrently, links between intolerance of uncertainty and later marijuana use may be insignificant due to the strong associations between intolerance of uncertainty and internalizing issues (Carleton, 2016). In addition, the stability of continued marijuana use was high, suggesting that emerging adults who use marijuana continue to use it at similar levels over time. Future work should examine possible mechanisms through which intolerance of uncertainty predicts emerging adult marijuana use.

Positive sibling relationship quality moderated the association between intolerance of uncertainty and later marijuana use in emerging adults when previous levels of marijuana use was not included. Accounting for the continuity of marijuana use may obfuscate potential moderators given fairly stable use patterns. However, omitting autoregressive effects may assist in identifying potential influences that can be further explored, such as sibling relationship quality. Emerging adults who perceived lower positive sibling relationships from younger siblings showed a mild positive association between intolerance of uncertainty and marijuana use when baseline levels were omitted, such that higher intolerance of uncertainty associated with higher marijuana use in the context of poor sibling relationships. Our findings suggest emerging adulthoods may use more marijuana to cope with uncertainty when their sibling relationships are lacking, likely as siblings may provide supports that may help to combat stress (Rogers et al., 2018). As siblings can influence marijuana use in adolescence (Defoe et al., 2023), it is expected that their continued communication throughout emerging adulthood plays a role in their use decisions. Given that stressors can promote marijuana use (Epstein et al., 2015), supportive sibling interactions that reduce stress may act as a potential resiliency factor to lessen marijuana use. Future work should continue to examine the effect that siblings have on marijuana use, while accounting for prior and current use.

#### Health risk substances

Though prior work suggests that intolerance of uncertainty predicts health risk substances, such as opioid use (Garami et al., 2017), this study did not find this link. Health risk substances come with their own degree of uncertainty such that access to and use of these substances can depend on availability of funds and finding sources/dealers (Doyle & Huskinson, 2023). As the current sample included college attending emerging adults, they may have more difficulty affording and attaining these substances given more instability in their lives, compared to older and more established adults. Additionally, college students may be less likely to use some of the substances included in health risk substances (i.e., opioids and injectable drugs), which may contribute to lessened use. Future work should explore the link between intolerance of uncertainty and health risks substances in samples with greater use, and compare across adult age groups, to identify whether these effects exist in other populations.

Positive sibling relationship quality served as a moderator between intolerance of uncertainty and later health risks substances. Poor sibling relationship quality negatively impacted the link between intolerance of uncertainty and later health risks substances, suggesting less positive sibling relationships can reduce health risks substance use as intolerance of uncertainty increases. As noted previously, sibling relationships can be both positive and negative, and there are times in which siblings contribute to one another’s delinquency (Whiteman et al., 2017). However, having fewer positive relationships with younger siblings may be beneficial as it reduces engagement with these more potentially harmful substances, contrary to other studies (Kothari et al., 2014). Emerging adults may perceive more uncertainty related to recreational substance use (Doyle & Huskinson, 2023), with lacking sibling support potentially contributing to negative perceptions of use when intolerance of uncertainty is high. Thus, emerging adults may decrease their health risk substance use following high intolerance of uncertainty when they perceive poorer relationship quality with younger siblings to lessen those negative perceptions. Though, effects observed in this study may vary among larger samples of substance using emerging adults. The field would benefit greatly from research on the influence of siblings on specific health risks substances initiation and continued use in emerging adulthood.

#### Alcohol use

Intolerance of uncertainty has been known to predict alcohol use in emerging adulthood (Oglesby et al., 2015), though most effects have been cross-sectional. Intolerance of uncertainty did not predict later alcohol use, potentially as the prevalence of drinking behaviors among college students often involves contextual and social factors, especially as drinking culture is pervasive at universities (Schulte & Li, 2022). As emerging adult drinking behaviors can vary based on context, intolerance of uncertainty may not directly influence change in drinking behaviors for general college students. Examining multiple individual and contextual factors when assessing emerging adult drinking behaviors may better assist in describing how intolerance of uncertainty and alcohol use are linked, such that emerging adult internalized experiences may interact with their environment to influence drinking behaviors.

Positive sibling relationship quality did not moderate the association between intolerance of uncertainty and later alcohol use, although it did directly predict lessened alcohol use. As emerging adults report relatively stable alcohol consumption across time (Schulte & Li, 2022), shared relationships with younger siblings may not serve as a prominent source of influence on drinking behaviors. Though siblings can promote and exacerbate alcohol use in emerging adulthood (Kothari et al., 2014), these findings suggests that younger sibling relationship quality does not affect the link between intolerance of uncertainty and later alcohol use, possibly due to the complexities of drinking behaviors of college attending emerging adults. However, findings indicated better sibling relationships reduced the amount of alcohol consumed later on. Though these effects have been seen before from older to younger siblings in emerging adulthood (Kothari et al., 2014), the influence of younger siblings on older siblings drinking behaviors are unique. This suggests that siblings continue to have a beneficial impact on one another’s drinking behaviors across emerging adulthood, with bidirectional influences present. Future work should continue to examine the effect siblings may have on one another’s drinking behaviors in emerging adulthood, while accounting for internalizing issues, to better describe what effects may exists and how drinking trajectories are impacted by sibling relationships.

### Limitations and future directions

A central strength of this study is its two-cohort longitudinal design and inclusion of multiple outcomes for psychopathology; however, several limitations should be considered. The sample includes only college students who primarily identified as women. Inclusion of non-college attending emerging adults would bolster our understanding of development by providing representation across additional trajectories of those pursuing adult roles (Arnett, 2015). In addition, women often report greater internalizing issues and lower substance use compared to men (Essau et al., 2010), which may have influenced reports of depression and anxiety and limited the responses for substance use behaviors as only 17 men were included in the study. Furthermore, participants were not formally evaluated for mental health diagnoses. To enhance our understanding of how intolerance of uncertainty is linked with psychopathology, future work should include formal diagnoses and more detailed experiences of mental health and regular substance using populations.

Another limitation to consider is that positive sibling relationship quality was only captured through emerging adult older sibling perceptions of warmth and hostility, leaving younger adolescent sibling perceptions unaccounted for. Through a combined measure of both warmth and hostility aids in more fully capturing the dynamic nature of sibling relationships, there are additional features which should be explored. Inclusion of contact frequency, emotional closeness, dyadic responses, and specific supportive behaviors, would help to further conceptualize the sibling relationship dynamic. Examining how sibling relationship features function during periods of stress may offer valuable insight and relevant treatment targets to support emerging adult psychopathology. In addition, this study had high rates of attrition, reducing the initial sample to 21% of its original size. Despite this limitation, the sample size still afforded enough power to detect direct and moderated effects using longitudinal structural equation modeling. Further, two-cohort design minimized cohort effects, which is especially useful given variation in intolerance of uncertainty for adolescents transitioning into emerging adulthood following the COVID-19 pandemic. This study is among few that have examined intolerance of uncertainty as a predictor of later psychopathology (Sahib et al., 2024) and contributes to our understanding of how sibling relationships may influence psychopathology.

### Implications

This study demonstrates that emerging adult intolerance of uncertainty is associated with later perceptions of depression and anxiety, as well as substance use behaviors, when accounting for sibling relationship quality. Positive sibling relationship quality with younger siblings can serve as both a protective and risk factor for emerging adult substance use following experiences of intolerance of uncertainty. As many emerging adults report sustained communication with their siblings (Lindell et al., 2015), relationships with adolescent siblings appear to remain salient for emerging adults. Though mental health often declines during early emerging adulthood, supportive sibling relationships can provide an ameliorating effect to reduce mental health issues (Finan et al., 2018). Promoting sibling relationships in emerging adulthood may benefit a host of adaptive reactions to stress and assist in the maintenance of one of the longest relationships across their lifespan (Jensen et al., 2022). As emerging adults report greater experiences with depression, anxiety, and substance use (Epstein et al., 2015; Finan et al., 2018), the usefulness of sibling relationships may be especially important in predicting and managing emerging adult substance use. These findings highlight the importance that sibling relationships continue to have on emerging adult externalizing behaviors.

## Data Availability

The data, analysis code, and materials used in this study are not openly available but are available upon request to the corresponding author. No aspects of the study were pre-registered.
